# Clinical outcomes of early versus standard liver transplantation in patients with alcohol-related liver disease: a meta-analysis

**DOI:** 10.1007/s00423-026-04310-0

**Published:** 2026-10-02

**Authors:** Solonas Symeou, Kevin Lee Boon Chun, Emmanouil Sinakos, Georgios Tsoulfas, Austin D. Schenk

**Affiliations:** 1 School of Medicine, University of Ioannina, Ioannina, 45500 Greece; 2 School of Medicine, Cardiff University, Cardiff, CF14 4YS Wales, UK; 3 Department of Internal Medicine, Hippokratio Hospital, Aristotle University of Thessaloniki, Thessaloniki, 54642 Greece; 4 Department of Transplantation Surgery, Center for Research and Innovation in Solid Organ Transplantation, School of Medicine, Aristotle University of Thessaloniki, Thessaloniki, 54642 Greece; 5 Department of Surgery, The Ohio State University Wexner Medical Center, Columbus, OH 43210 USA

**Keywords:** Liver transplantation, Alcohol-related liver disease, Alcoholic hepatitis, Early transplantation, Standard transplantation, Alcohol abstinence

## Abstract

**Background:**

A 6-month abstinence period before listing patients with alcohol-related liver disease (ALD) for liver transplantation (LT) is the current standard practice at many transplant centers worldwide. Its prognostic validity is, however, debated because it has not been prospectively validated, and because many ALD patients do not survive long enough to make it onto the waitlist.

**Methods:**

A meta-analysis was conducted in accordance with the guidelines put forth by Cochrane and the Preferred Reporting Items for Systematic Reviews and Meta-Analyses (PRISMA). Three databases (PubMed/Medline, Scopus and Cochrane Library) were searched to identify studies comparing early (eLT) and standard LT (sLT).

**Results:**

Twelve studies comparing eLT and sLT for ALD were included, encompassing 2323 patients with ALD. Mortality [Risk ratio (RR) = 0.94, 95% Confidence Interval (CI): 0.73–1.22] and patient survival [Hazard Ratio (HR) = 1.10, 95% CI: 0.83–1.45] did not differ significantly between eLT and sLT. Any alcohol relapse was more frequent following eLT but did not reach significance in the primary analysis (RR = 1.37, 95%CI: 0.95-2.00). This association became statistically significant on leave-one-out and subgroup analyses. Early LT patients had a higher risk of acute graft rejection (RR = 1.22, 95% CI: 0.98–1.52) but comparable graft loss (RR = 0.97, 95% CI: 0.74–1.27). Post-operative risk of infection did not differ significantly (RR = 1.19, 95% CI: 0.80–1.77), but eLT was associated with longer hospitalization [Mean Difference (MD) = 5.24 days, 95% CI: 0.15–10.33].

**Conclusions:**

Early LT offers survival similar to sLT and is a reasonable option when medical therapy fails. However, the equivocal evidence of higher morbidity and alcohol relapse, and the longer hospitalization, support careful multidisciplinary selection and close post-transplant surveillance, particularly among recipients with alcohol-related hepatitis.

**Supplementary Information:**

The online version contains supplementary material available at https://doi.org/10.1007/s00423-026-04310-0.

## Introduction

Alcohol-related liver disease (ALD) is the leading cause of chronic liver injury and liver-related mortality globally [1, 2]. A recent epidemiological study estimates the global prevalence of ALD to be 4.8% with alcohol-related cirrhosis (AC) accounting for almost one third (32.9%) of cases at the time of diagnosis [2]. Although only 15–20% of heavy drinkers will develop cirrhosis during their lifetime, alcohol is implicated in more than one quarter of cirrhosis-related deaths, while per capita alcohol consumption is projected to reach 7.6 L per year by 2030, foreshadowing a continuous rise in the prevalence of end-stage liver disease (ESLD) [1, 3, 4].

Transplant activity reflects the growing burden of ALD. According to the Organ Procurement and Transplantation Network (OPTN) and the Scientific Registry of Transplant Recipients (SRTR) annual data report (2023), ALD has become the most prominent indication for adult liver transplantation (LT) in the US, accounting for 41.1% of all recipients, ahead of metabolic dysfunction-associated steatohepatitis (20.3%) and viral-associated hepatitis (4.2%) [5]. Similarly, ALD dominates the waitlist, comprising 34.9% of the listed adult candidates with alcohol-related hepatitis (AH) representing an additional 4.1% [5]. Despite this rapid expansion of the recipient pool, outcomes remain favorable, with patient survival rates of 91% and 76% at 1 and 5 years post-LT, respectively [6].

Candidate selection in ALD has remained a matter of debate. Unlike other transplant indications, a majority of centers worldwide still mandate at least 6 months of documented alcohol abstinence prior to listing [7–9]. This “6-month rule” is based on three traditional arguments: (a) prolonged pre-transplant sobriety likely predicts sustained post-transplant abstinence from alcohol; (b) a sufficient abstinence interval allows the native liver to recover and potentially precluding the need for LT; (c) scarce grafts should be preferentially allocated to patients least likely to damage them through recidivism [9]. None of these, however, were derived from prospective comparative data or formally validated, and the rule has therefore been criticized as arbitrary. Its direct clinical consequences could be devastating, with decompensated AC carrying a 1-year mortality approaching 50% while patients with severe AH non-responsive to corticosteroid therapy have a 6-month survival rate less than 25% [10, 11]. Consequently, a considerable proportion of ALD patients die within the mandated abstinence window before becoming eligible for transplantation.

Over the past decade, three major studies have attempted to bridge this gap. In particular, the Franco-Belgian cohort of Mathurin et al., the American Consortium of Early Liver Transplantation for Alcoholic Hepatitis (ACCELERATE-AH) and the first prospective controlled trial (QuickTrans), all showed that in carefully selected patients, early LT (eLT; <6 months of abstinence) yields favorable post-transplant survival with acceptable rates of alcohol relapse [12–14]. However, available evidence remains heterogeneous and limited. Existing cohorts vary substantially in terms of patient selection criteria and are often constrained by modest sample sizes. In addition, previous meta-analyses have relied extensively on non-comparative cohorts and conference abstracts, utilizing a smaller number of comparative studies, and have focused almost exclusively on patient survival and alcohol relapse. Other perioperative outcomes such as post-LT infection rates, graft rejection and length of hospitalization remain unexplored, while the analyses pooled more heterogeneous ALD populations [15–18]. Therefore, we performed an updated meta-analysis of all available comparative-only studies evaluating eLT and standard LT (sLT; >6 months of abstinence prior to LT) for ALD, expanding the range of outcomes and incorporating a subgroup analysis restricted to patients with AH. By concentrating a larger body of evidence and assessing a broader spectrum of outcomes, this study aims to provide the most comprehensive and contemporary evidence for the optimal timing of LT in adults with ALD, encouraging future refinements in patient selection criteria and clinical guidelines.

## Methods

This review was conducted in accordance with the recommendations put forth by Cochrane Collaboration and the Preferred Reporting Items for Systematic Reviews and Meta-Analyses (PRISMA) guidelines [19, 20]. The study protocol was prospectively registered in the International Prospective Register of Systematic Reviews (PROSPERO) under the registration number CRD420261382212 on April 28, 2026.

### Eligibility criteria

Studies were eligible for inclusion if they met all of the following criteria: (a) a direct head-to-head comparison of eLT with sLT within a comparative design (randomized trial, prospective or retrospective cohort); (b) enrolment of adult recipients undergoing LT for ALD, including AC and/or AH as defined by standard clinical, biochemical and/or histological criteria within each study; (c) reporting of at least one of the pre-specified outcomes of interest; and (d) publication in English. Conversely, studies were excluded if they did not report any of the outcomes of interest, lacked a direct comparator arm (e.g. case series), were available only as conference abstracts or letters to the editor, or represented study protocols without available results.

### Outcomes

The primary endpoints were mortality and any alcohol relapse. The latter was defined as any documented post-transplant alcohol consumption, irrespective of severity, frequency, or subsequent re-establishment of abstinence. Secondary outcomes included patient survival, graft loss, acute graft rejection, postoperative infection rates (bacteremia), and length of hospitalization (LOS).

### Search strategy and data extraction

We systematically searched PubMed/MEDLINE, Scopus and the Cochrane Library on April 30, 2026 for relevant studies, using the following keywords: “alcohol liver disease”, “alcoholic hepatitis”, “alcoholic liver”, “alcohol-associated liver disease”, “severe alcoholic hepatitis”, ALD, SAH, AH, AC, “liver transplantation”, “liver transplant”, LT, early, “early liver transplantation”, eLT, “preemptive liver transplantation”, late, “late liver transplantation”, “standard liver transplantation”, “standard”, “sLT”, sobriety, delayed, abstinence, “6-months” and sober. The full search strategy is provided in online supplementary Table 1.

Two authors (S.S., K.L.B.C.) independently conducted the literature search and screened the titles and abstracts of the retrieved records. Studies progressing to full-text review were then assessed against the predefined inclusion and exclusion criteria. Data extraction from the original publications was independently performed by the same two authors, and any disagreements were resolved by consensus.

### Risk of bias assessment

Risk of bias (RoB) was independently assessed by two authors (K.L.B.C., S.S.) using the Risk of Bias In Non-randomized Studies of Intervention (ROBINS-I) tool. ROBINS-I evaluates studies across seven domains: confounding, selection of participants, classification of interventions, deviations from intended interventions, missing data, measurement of outcomes, and selection of the reported result [21]. Each study received a domain-level and overall judgement of low, moderate, serious, or critical risk, with any disagreements resolved by consensus.

### Statistical analysis

Risk ratios (RR) and mean differences (MD) with 95% confidence intervals (CIs) were calculated for all binary and continuous outcomes, respectively. For time-to-event analyses, hazard ratios (HRs) were extracted directly from the original publications, or when only Kaplan Meier (KM) curves were available, reconstructed using the IPDfromKM algorithm followed by Cox proportional hazards regression [22]. The proportional hazards assumption was assessed for all studies providing HRs, using Schoenfeld residuals. Adjusted estimates were preferred over unadjusted ones when both were reported. A random-effects model was applied to all outcomes to account for anticipated clinical and methodological heterogeneity arising from differences in patient selection criteria, established diagnosis, sample sizes and outcome definitions across studies. Binary outcomes were pooled using the Mantel-Haenszel method, given its suitability for the synthesis of sparse data, whereas the inverse of variance method was used for the LOS, in line with the Cochrane recommendations. The between-study heterogeneity (tau^2^=t^2^) was estimated using the restricted maximum likelihood (REML) method, and the corresponding 95% CIs were derived using the Hartung-Knapp/Sidik-Jonkman (HKSJ) adjustment. Statistical heterogeneity was further assessed using the I^2^ statistic and Cochran’s Q test, with I^2^ values > 30% and p-values < 0.10 considered indicative of substantial heterogeneity. A 95% prediction interval (PI) was additionally calculated for each pooled estimate. All statistical analyses were performed using R software, V.4.5.3 (R Foundation for Statistical Computing, Vienna, Austria).

### Sensitivity analysis and publication bias

Leave-one-out (LOO) analyses were performed to identify studies exerting a disproportionate influence on the pooled estimates, as well as to explore potential sources of heterogeneity. The LOO analysis was performed by sequentially omitting one study at a time and reanalyzing the remaining dataset, for outcomes with I^2^ >30%. Given the clinical heterogeneity of patients with ALD, particularly with respect to underlying diagnosis and disease severity, subgroup analyses restricted to patients with AH were conducted for outcomes reported by at least three studies. Publication bias was visually assessed using funnel plots for all outcomes. Egger’s test was not performed because fewer than ten studies contributed to each.

## Results

### Study selection

**Fig. 1:**
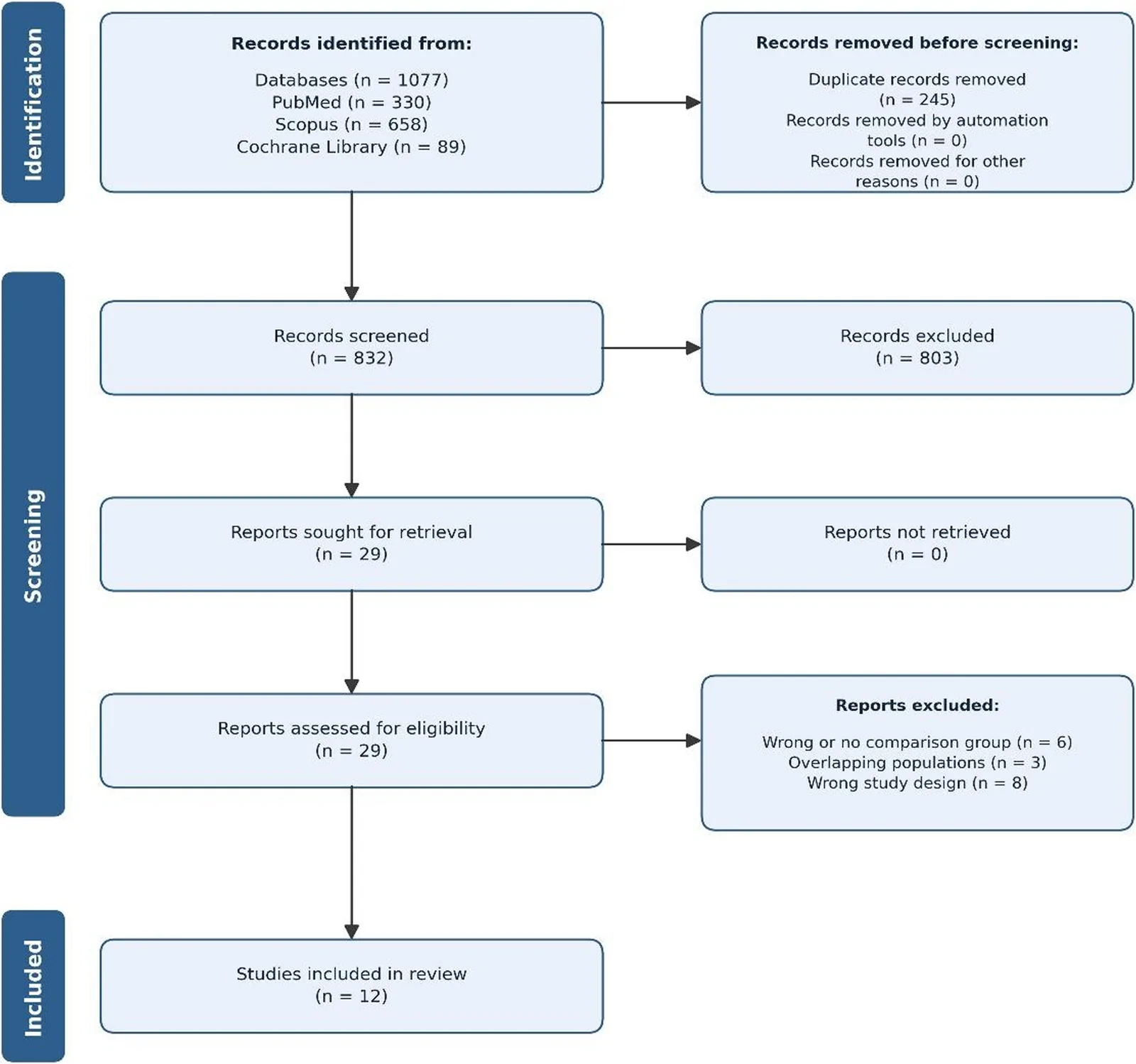
PRISMA flow diagram of study screening and selection

The initial search yielded 1077 records. After removal of 245 duplicates and exclusion of 803 records that did not meet the eligibility criteria, 29 articles proceeded to full-text assessment. Of these, 12 were ultimately included in the quantitative analysis, with care taken to avoid overlapping study populations. The complete study selection process is illustrated in Fig. 1.

### Study characteristics

The characteristics of the included studies are summarized in Table 1. Of the twelve included studies, ten were retrospective cohorts, one was a non-randomized trial, and one was a prospective cohort with historical controls [14, 23–33]. Study periods spanned three decades, from 1988 to 2023, although most data were derived from studies conducted after 2015 (Table 1). Nine studies originated from the United States and were predominantly single-center experiences, with one multicenter analysis based on data from the OPTN registry [33]. The remaining three studies came from Canada, Taiwan, and a Franco-Belgian consortium involving centers in France and Belgium [14, 25, 28].

**Table 1:** Baseline characteristics of the included studies; eLT= Early liver transplantation; sLT= Standard liver transplantation; AH= Alcohol-related hepatitis; AC= Alcohol-related cirrhosis; OPTN= Organ Procurement and Transplantation Network; NR = Not reported; *=Mean

Author & Year	City, Country	Study Design	Study Period	Total number of patients	Male sex, *N* (%)	Median Follow-up
Andacoglu et al., 2021	New York, USA	Retrospective Cohort	2015–2019 (AH group) 2010–2019 (AC group)	eLT: 15 sLT: 46 Total: 61	eLT: 8 (53.3%) sLT: 32 (69.5%)	365 days
Asgharpour et al., 2023	Richmond, USA	Retrospective Cohort	2018–2022	eLT: 85 sLT: 86 Total: 171	eLT: 51 (60%) sLT: 60 (70%)	365 days
Carrique et al., 2021	Toronto, Canada	Prospective Cohort with Historical Controls	2018–2020 (eLT group) 2016–2018 (Controls)	eLT: 44 sLT: 111 Total: 155	eLT: 28 (63.6%) sLT: 84 (75.7%)	339 days*
Fu et al., 2025	Stanford, USA	Retrospective Cohort	2018–2022	eLT: 58 sLT: 111 Total: 169	eLT: 42 (72.4%) sLT: 90 (81.0%)	NR
Herrick-Reynolds et al., 2021	Baltimore, USA	Retrospective Cohort	2012–2020	eLT: 88 sLT: 75 Total: 163	eLT: 59 (67%) sLT: 49 (65%)	701 days
Hung et al., 2026	Taoyuan, Taiwan	Retrospective Cohort	2002–2023	eLT: 28 sLT: 6 Total: 34	eLT: 26 (92.8%) sLT: 6 (100%)	740 days
Legg et al., 2024	Maryland, USA	Retrospective Cohort	2015–2021	eLT: 130 sLT: 98 Total: 228	eLT: 73 (56%) sLT: 73 (74%)	365 days
Meinders et al., 2025	Houston, USA	Retrospective Cohort	2019–2021	eLT: 99 sLT: 84 Total: 183	eLT: 59 (59.6%) sLT: 60 (71.4%)	644 days
Musto et al., 2024	Madison, USA	Retrospective Cohort	2010–2020	eLT: 110 sLT: 234 Total: 344	eLT: 75 (68.2%) sLT: 170 (72.6%)	1680 days
Shakil et al., 1997	Pittsburgh, USA	Retrospective Cohort	1988–1996	eLT: 9 sLT: 425 Total: 434	eLT: 8 (88.9%) sLT: NR	1705 days
Louvet et al. 2022	Europe (Franco-Belgian)	Non-randomized Controlled Trial	2012–2016	eLT: 68 sLT: 93 Total: 161	eLT: 46 (67.7%) sLT: 68 (73.1%)	730 days
Singal et al. 2012	Multicenter (OPTN data), USA	Retrospective Cohort	2004–2010	eLT: 55 sLT: 165 Total: 220	eLT: 42 (76.4%) sLT: 126 (76%)	NR

Across all the studies, a total of 2,323 patients were included, of whom 789 underwent eLT and 1,534 sLT. Study sample sizes varied considerably, ranging from 34 patients in Hung et al. to 434 patients in Shakil et al. [28, 32]. Male sex predominated in both groups, accounting for approximately 53% to 93% and 65% to 100% of the eLT and sLT patients, respectively (Table 1). Median follow-up time was reported in nine of the twelve studies, and it ranged from 365 to 1,705 days [23, 32].

### Clinical characteristics

The clinical characteristics of the included studies are summarized in Table 2. The diagnostic approach to ALD was reasonably consistent across studies, although some variation was present. Eight of the twelve studies relied on clinical criteria, with biopsy or explant histology examination used for confirmation in three [14, 25, 27] and clinical diagnosis alone in five [24, 26, 28, 30, 31]. Of the remaining four studies, one defined AH retrospectively on explant histology, two used registry-based definitions such as the primary listing indication recorded in the United Network for Organ Sharing (UNOS) [29, 32, 33]. Six of the twelve studies included data on patients with AH in the eLT group, allowing subgroup analyses to be performed [14, 23, 24, 28, 32, 33]. As expected, the difference in pre-transplant alcohol abstinence between eLT and sLT was substantial. Among studies explicitly reporting this variable, median pre-LT abstinence from alcohol ranged from 46 to 75 days in the eLT group, compared with 388 to 580 days in the sLT arm [26, 29, 30]. This distinction, which underlies the separation between the two transplantation strategies, was consistently maintained across geographic settings and study periods.

**Table 2:** Clinical characteristics of the included studies; ALD= Alcohol-related liver disease; NIAAA= National institute on alcohol abuse and alcoholism alcoholic hepatitis consortia; AASLD= American association for the study of the liver diseases; AH= Alcohol-related hepatitis; SAH= Severe alcohol-related hepatitis; AC= Alcohol-related cirrhosis; eLT= Early liver transplantation; sLT= Standard liver transplantation; NR = Not reported; UNOS= United Network for Organ Sharing; *= Mean

Author & Year	Diagnosis	Type of donor	Pre-LT Abstinence (Median, days)	MELD at Listing (Median)	MELD at transplant (Median)	Time on waiting list (Median, days)
Andacoglu et al., 2021	AH was vaguely defined AC: NR	eLT: 100% Cadaveric sLT: 89.2% Cadaveric, 10.8% Living	NR	NR	eLT: 36 sLT: 39	NR
Asgharpour et al., 2023	SAH was defined by clinical criteria (NIAAA)	100% Cadaveric	*eLT: 132 *sLT: 703 *SAH subgroup: 58	NR	*eLT: 36 *sLT: 26	*eLT: 14 *sLT: 83
Carrique et al., 2021	SAH was defined by clinical criteria (NIAAA) and biopsy AC was vaguely defined	eLT: 91% Cadaveric, 9% Living sLT: NR	*eLT: 122 *sLT: NR	*eLT: 26 *sLT: 19.2	*eLT: 27.1 *sLT: 20.3	*eLT: 69.8 *sLT: 180.8
Fu et al., 2025	ALD was defined by clinical criteria	100% Cadaveric	eLT: 75 sLT: NR	NR	eLT: 39 sLT: 34	NR
Herrick-Reynolds et al., 2021	AH was defined by clinical criteria (AASLD) and explant pathology AC: NR	eLT: 100% Cadaveric sLT: 85% Cadaveric, 15% Living	eLT: 66.5 sLT: 481	eLT: 35 sLT: 20	eLT: 36 sLT: 24	eLT: 7 sLT: 77
Hung et al., 2026	SAH was defined by clinical criteria	eLT: NR sLT: NR	NR	NR	NR	NR
Legg et al., 2024	AC or AH listed as primary indication for LT within the UNOS database	100% Cadaveric	eLT: 46 sLT: 388	eLT: 36 sLT: 23	eLT: 37 sLT: 27	eLT: 6 sLT: 51
Meinders et al., 2025	AH was defined by clinical criteria	eLT: 100% Cadaveric sLT: 94% Cadaveric, 6% Living	eLT: 55 sLT: 580	NR	eLT: 38 sLT: 29	eLT: 5 sLT: 113
Musto et al., 2024	AH was defined by clinical criteria (NIAAA) AC was vaguely defined	eLT: 100% Cadaveric sLT: 99.6% Cadaveric, 0.4% Living	eLT: 61.5 sLT: 552	eLT: 38 sLT: 28	NR	eLT: 18 sLT: 192
Shakil et al., 1997	AH was defined by explant biopsy (retrospectively)	NR	NR	NR	NR	NR
Louvet et al. 2022	AH was defined by clinical criteria and explant biopsy AC: NR	NR	NR	NR	eLT: 31 s LT: 22	NR
Singal et al. 2012	AC or AH listed as primary indication for LT within the UNOS database	eLT : 100% cadaveric sLT: 100% cadaveric	NR	*eLT: 25.7 *sLT: 25.6	NR	*eLT: 67 *sLT: 145

Disease severity also followed a consistent pattern. Early LT recipients generally had higher Model for End-Stage Liver Disease (MELD) scores, with a median MELD at listing ranging from 35 to 38 compared with 20 to 28 for the sLT recipients [27, 31]. At the time of transplantation, median MELD scores ranged from 31 to 39 in the eLT group, compared with 22 to 39 in the sLT group, reflecting disease progression while on the waitlist [14, 26]. Waitlist time was, as expected, considerably shorter among patients undergoing eLT, with median values ranging from 5 to 18 days, compared with 51 to 192 days among sLT recipients [29–31].

Regarding donor type, all grafts allocated to the eLT arm were procured from deceased donors in eight of the studies (Table 2). One additional study reported cadaveric graft proportion exceeding 90%, whereas the remaining studies did not provide donor-type data [25]. In the sLT group, the proportion of living-donor transplantation ranged from 0.4% to 15% [27, 31].

### Primary outcomes

**Fig. 2:**
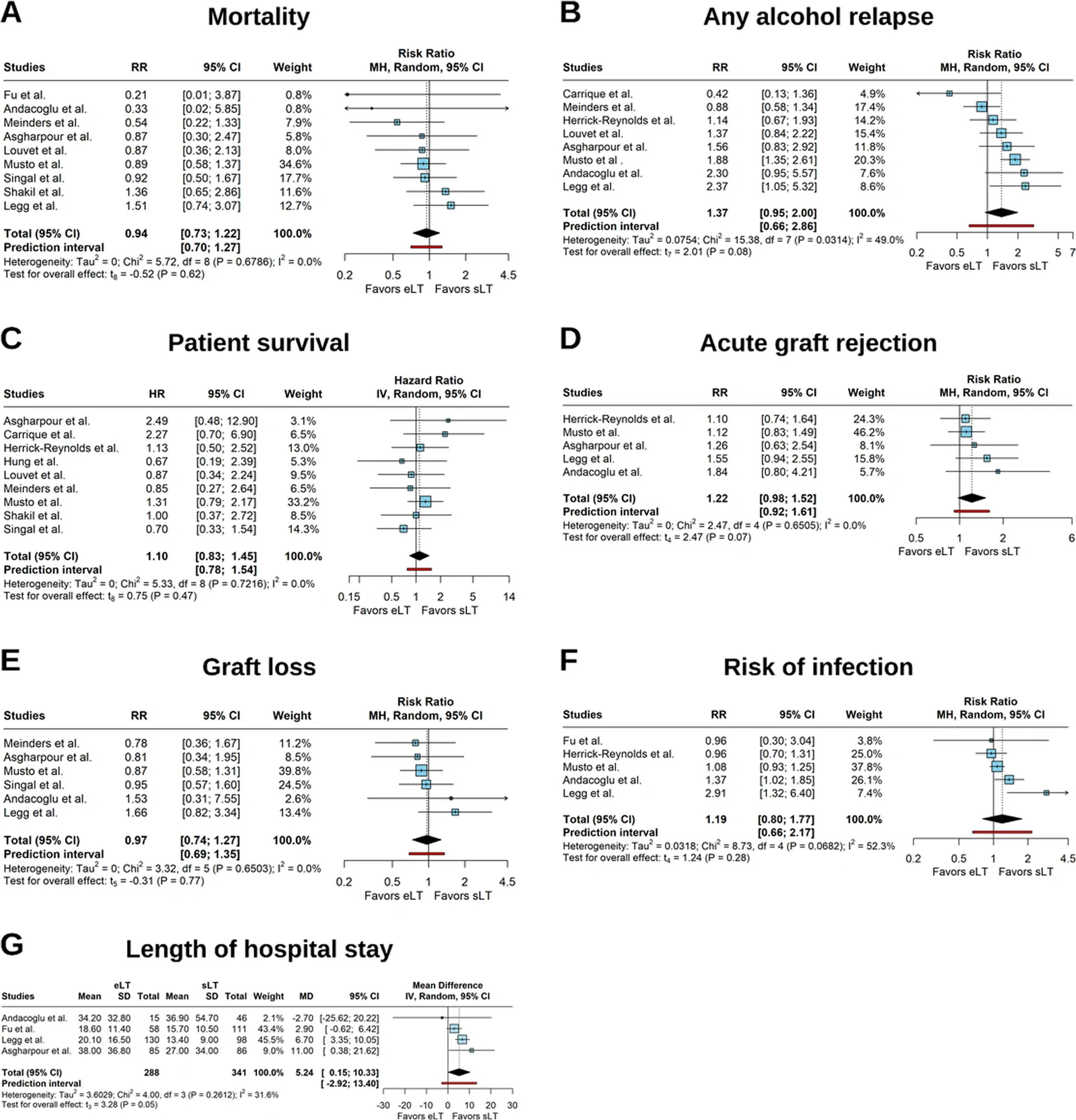
Forest plots of the pooled outcomes; RR= Risk ratio; HR= Hazard ratio; MD= Mean difference; CI= Confidence interval; eLT= Early liver transplantation; sLT= Standard liver transplantation; IV= Inverse of variance; MH= Mantel-Haenszel

Nine studies reported mortality. The pooled RR was 0.94 (95% CI:0.73–1.22; Fig. 2A), numerically favoring the eLT group but without reaching statistical significance, and importantly, with no heterogeneity across the studies (I^2^ = 0%, *P* = 0.68). Any alcohol relapse was more common among patients in the eLT (RR = 1.37, 95% CI: 0.95-2.00; Fig. 2B), although this too did not reach statistical significance. As a result of the variability in how this outcome was defined, significant heterogeneity was evident (I^2^ = 49%, *P* = 0.03).

### Secondary outcomes

Patient survival was available from nine studies, five providing direct HRs (three adjusted and two unadjusted) and four with HRs reconstructed from KM curves [14, 24, 25, 27, 28, 30–33]. The proportional hazards assumption was met in all studies. The pooled HR was 1.10 (95% CI: 0.83–1.45, I^2^ = 0%; Fig. 2C), with patients in the sLT group having a slightly longer survival, though the difference was not statistically significant. Acute graft rejection was reported in five studies and was more frequent in patients undergoing eLT, although this was not statistically significant (RR 1.22, 95% CI: 0.98–1.52, I^2^ = 0%; Fig. 2D). The risk of graft loss was evaluated in six studies and was not statistically different between the two groups (RR 0.97, 95% CI: 0.74–1.27, I^2^ = 0%; Fig. 2E). Post-operative infection was numerically more prevalent among patients undergoing eLT but without reaching statistical significance (RR 1.19, 95% CI: 0.80–1.77; Fig. 2F). Heterogeneity was substantial (I^2^ = 52.3%, *P* = 0.068) likely reflecting differences in the pre-operative hospitalization and infection status of patients, the site of infection, and the cultured microorganisms. Finally, eLT was associated with a significantly longer hospital stay, prolonged by a mean of 5.24 days compared to sLT (MD = 5.24 95% CI: 0.15–10.33, I^2^ = 31.6%; Fig. 2G).

### Risk of bias assessment

The RoB varied across the twelve included studies. None was judged to be at low overall bias, with confounding representing the principal concern in all studies, as each was rated at least moderate in Domain 1. Eight studies were judged to be at moderate overall bias and four at serious risk [23, 24, 29, 32]. Overall judgements closely reflected the assessment of Domain 1 (confounding), with the four studies rated at serious risk of confounding also receiving a serious overall bias judgement. Assessments across the remaining domains were generally more favorable. Domain 2 (selection of participants) was judged to be at low risk in 11 studies, with only Shakil et al. rated at moderate risk [32]. Domain 3 (classification of interventions) was rated as low risk in 11 studies, with Andacoglu et al. judged to be at moderate risk [23]. The same pattern was observed for Domain 7 (selection of the reported result).

**Fig. 3:**
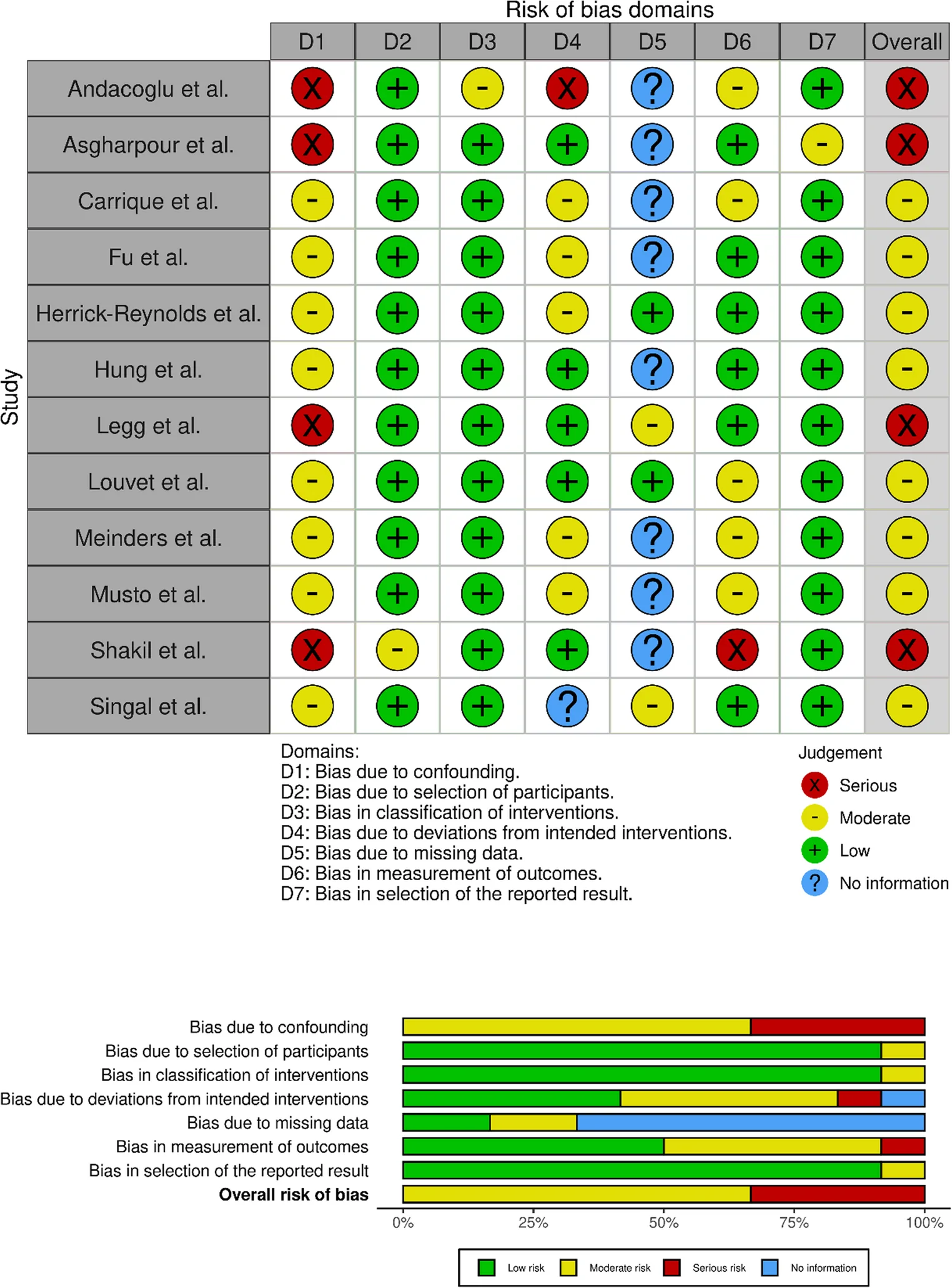
Risk of bias assessment

Domain 4 (deviations from intended interventions) showed greater variation, with five studies rated at low risk and five as moderate risk, while Andacoglu et al. was judged to be at serious risk and Singal et al. as providing no information [23, 33]. For Domain 5 (missing data), only two studies provided sufficient information to be rated as low risk and two as moderate, whereas the remaining eight provided insufficient information to permit a judgement and were therefore classified as having no information [14, 27, 29, 33]. In domain 6 (measurement of outcomes), six studies were judged to be at low risk, five at moderate and Shakil et al. at serious [32]. The complete RoB assessment is presented in Fig. 3.

### Sensitivity analyses

Leave-one-out analyses were performed for any alcohol relapse, infection risk, and LOS. For any alcohol relapse, the direction of effect remained consistent across all iterations, with recalculated RRs ranging from 1.26 to 1.54, consistently favoring sLT (Supplementary Fig. 1A). However, omission of either Carrique et al. or Meinders et al. resulted in a statistically significant pooled effect (RR = 1.45, 95% CI: 1.05–2.02; and RR = 1.54, 95% CI: 1.09–2.20, respectively) [25, 30]. These findings suggest that the statistical significance of the pooled estimate was sensitive to the inclusion of these two cohorts.

For infection risk, the direction of the summary estimate likewise remained stable across all iterations (RR: 1.10–1.33), although none achieved statistical significance. Heterogeneity decreased markedly from 52.3% to 1.7% following exclusion of Legg et al., suggesting that this study accounted for much of the observed between-study variability [29]. For LOS, the mean difference remained positive throughout. However, exclusion of Asgharpour et al., Andacoglu et al., or Legg et al., widened the 95% CI to include the null [23, 24, 29]. This suggests that the pooled LOS estimate is influenced by a subset of studies rather than being consistently supported across all cohorts. With respect to heterogeneity, omission of Fu et al. reduced I^2^ to 0% while preserving the statistical significance [26].

In the subgroup analysis restricted to patients with AH in the eLT group, four outcomes were reported by at least three studies (Supplementary Figs. 2A-D). Mortality was slightly higher in the eLT group (RR = 1.02, 95% CI: 0.69–1.50), whereas patient survival favored eLT numerically (HR = 0.80, 95% CI: 0.50–1.28). The estimate for graft loss was nearly unchanged from the primary analysis (RR = 0.97, 95% CI: 0.62–1.52), and none of these three outcomes reached statistical significance. Any alcohol relapse was the only endpoint, showing statistically significant difference, with a higher risk among patients undergoing eLT (RR = 1.56, 95% CI: 1.08–2.26). Despite the fact that no statistical heterogeneity was observed for any of the outcomes (I^2^ = 0%), the corresponding prediction intervals, including that for any alcohol relapse, crossed the null.

### Publication bias

Funnel plots were constructed for all outcomes (Supplementary Fig. 3A-G). Notably those for any alcohol relapse, acute rejection and infection risk, appeared visually asymmetric, raising the possibility of small-study or publication bias (Supplementary Figs. 3B, 3D, 3 F). Asymmetry was less evident for mortality, patient survival and graft loss, with studies distributed roughly symmetrically on either side (Supplementary Figs. 3A, 3 C, 3E). These observations should be interpreted cautiously, as the limited number of studies precludes any reliable distinction between publication bias, true between-study heterogeneity, and chance.

## Discussion

In this meta-analysis of 2,323 patients, we compared early LT with the standard “6-month” approach in patients with ALD. The main findings from the pooled analyses were the following: (a) mortality did not differ significantly between the two groups; (b) no significant difference was observed in patient survival; (c) any alcohol relapse was more frequent following eLT, although this finding should be interpreted cautiously, as the association was non-significant in the primary analysis but became statistically significant in the LOO analysis and the AH analysis. Notably, this trend was not accompanied by an increased risk of graft loss; and (d) eLT was associated with numerically higher rates of acute rejection and infection. Apart from a significantly longer LOS (MD = 5.24 days) none of these differences reached statistical significance.

Two recent meta-analyses have addressed this comparison and similarly reported no significant difference in mortality between eLT and sLT among patients with ALD [15, 17]. This consistency is notable given that the evidence base in our study was assembled differently, providing further support for the existing evidence despite differences in inclusion criteria, while allowing for a more precise interpretation. We included only comparative studies and screened for overlapping populations to ensure that each pooled estimate was based on direct comparisons without duplication of patients. Survival was analyzed as a time-to-event outcome thereby accounting for censoring and differences in follow-up duration across studies.

The populations examined also differed. One previous meta-analysis presented as AH-specific has included patients undergoing eLT for AC [17]. In the present study, we therefore defined the population of interest more broadly as ALD and restricted the prespecified subgroup analyses to patients with AH, for whom the debate surrounding pre-transplant abstinence is particularly relevant. Finally, acute graft rejection, post-transplant infection and LOS have not previously been pooled, despite their potential to reflect the greater clinical acuity of eLT recipients and their relevance to postoperative monitoring and the resources required to support an eLT program [15–18].

Survival is the outcome against which any abstinence requirement must ultimately be judged, given the rapidly fatal course of untreated or medically refractory ALD [10, 11]. The survival benefit of eLT over medical therapy for ALD is well established in the literature: 77% versus 23% in the multicenter cohort by Mathurin et al., 89% vs. 11% in a similar US-based study by Im et al., and approximately four-fold higher two-year survival rate in a subsequent Franco-Belgian study [12, 14, 34]. Our analysis addresses the next question, whether this survival benefit is preserved when eLT is compared not with medical therapy, but with conventional transplantation.

We found no significant difference between the two groups in either mortality or patient survival, with no observed statistical heterogeneity across the cohorts spanning three decades and five countries. Similar findings were observed in the subgroup of patients with AH, suggesting that the absence of a detectable survival difference extends to this clinically more severe population in whom the debate surrounding pre-transplant abstinence is particularly relevant. This consistency across cohorts should not, however, be interpreted as evidence that future studies will necessarily yield similarly concordant results. Prediction intervals for both mortality and patient survival were wide, indicating that future studies could plausibly produce estimates favoring either strategy. This is not unexpected, given that cohorts differ in several factors likely to influence outcomes, including the stringency of candidate selection, MELD score at transplantation, the proportion of recipients with AH rather than AC, and other institutional protocols governing listing and post-transplant care.

The absence of a survival difference is biologically plausible given the extent of the underlying disease. Liver injury in ALD is driven in part by a self-perpetuating innate immune response initiated by Kupffer cell activation and amplified through a cascade of pro-inflammatory cytokines including TNF-α, IL-1β, IL-6, IL-8 [35–37]. Once these processes have progressed to liver failure, alcohol abstinence alone may be insufficient to reverse the underlying hepatic dysfunction, leaving transplantation as the only potentially curative-intent treatment. Following transplantation, outcomes are therefore likely to depend not only on disease biology but also on the rigor of candidate selection. This is relevant in chronic manifestations of ALD, such as AC, where outcomes may be influenced by the careful assessment of both clinical and psychosocial factors [25, 38–40].

If survival outcomes do not support a mandatory abstinence interval, alcohol relapse remains one of the principal concerns regarding eLT, given its reported association with graft injury and increased mortality [41]. Although the primary pooled estimate did not reach statistical significance, any alcohol relapse was numerically more frequent among eLT recipients. This is broadly compatible with the transplantation literature, in which rates of return to alcohol use range from 22% to 34% [14, 42]. Additionally, this finding was sensitive to study composition, as omission of either of two studies in the LOO analysis shifted the pooled estimate to statistical significance, underscoring the uncertainty surrounding the primary result [25, 30]. A higher relapse rate following eLT is also biologically and behaviorally plausible, as the shortened interval to listing provides less time for the pre-transplant drinking behavior to be observed. The subgroup analysis restricted to patients with AH in the eLT arm, which demonstrated a significantly higher risk of alcohol relapse compared to sLT (RR = 1.56, 95% CI: 1.08–2.26), lends further support to this observation.

The clinical significance of this finding, however, should be interpreted in the light of two considerations. First, the higher relapse risk observed among eLT recipients was not accompanied by any significant increase in graft loss in either the primary or in the subgroup analyses, consistent with findings from previous analyses [15–17]. Second, although pre-transplant abstinence shorter than 6 months has been identified as a predictor of relapse in some studies, its predictive accuracy and reproducibility remain inconsistent, and a fixed interval captures only one component of a multifactorial risk profile [43–46]. Moreover, the prediction intervals for any alcohol relapse crossed the null in both the primary and subgroup analyses, indicating that the direction and magnitude of the association may vary across future clinical settings.

Importantly, the fact that this outcome reached statistical significance among patients with AH suggests that findings derived from the broader ALD population, particularly those regarding post-LT alcohol use, should not be extrapolated to this subgroup without qualification. In these patients, more rigorous selection and closer post-transplant surveillance may be more appropriate than withholding eT or imposing longer mandatory abstinence periods [47, 48].

Such selection may be structured using validated psychosocial instruments. The Stanford Integrated Psychosocial Assessment for Transplantation (SIPAT) and the Sustained Alcohol Use Post-Liver Transplant (SALT) score may help structure psychosocial assessment before eLT. SIPAT evaluates treatment readiness, social support, psychosocial stability, and substance use history, whereas SALT was developed to identify patients at low risk of sustained alcohol use after transplantation [49, 50]. SIPAT currently has stronger supporting evidence, with higher scores associated with harmful relapse irrespective of abstinence duration, whereas SALT has demonstrated less consistent performance [51]. However, neither tool reliably predicts relapse at the individual level, and both have demonstrated low positive predictive values in direct comparison [52].

These observations highlight three priorities for future research on alcohol relapse. First, the associations require confirmation in larger prospective cohorts, as the available evidence is based on a limited number of comparative studies and remains sensitive to study composition and sensitivity analyses [47]. Second, SIPAT, SALT and similar instruments should be prospectively evaluated, further improved and externally validated, particularly in recipients with AH, in whom candidate selection carries particularly important consequences and a fixed abstinence interval may be less informative [52]. Third, relapse ascertainment should be standardized across institutions. The studies included in this analysis documented recidivism using varying methods, including interviews, self-report and biomarker testing [14, 24, 25]. Uniform reporting of any alcohol use, isolated slips and sustained drinking as distinct outcomes, supported where possible by objective testing such as phosphatidylethanol (PEth), would allow this question to be addressed with greater confidence than the current literature permits [24, 52].

Unlike relapse, which may be mitigated through careful patient selection and follow-up, the early risks after eLT largely reflect the condition in which recipients reach transplantation [53]. Across the included cohorts, eLT recipients had higher MELD scores both at listing and at transplantation and substantially shorter waitlist times, often proceeding to surgery while acutely decompensated and with limited opportunity for physiological or nutritional optimization.

The higher infection risk may reflect the impaired immune function and disrupted gut barrier characteristic of these patients, whereas the borderline increase in acute graft rejection may relate to the difficulty of balancing immunosuppression against concurrent infection and renal impairment [35, 54]. Neither outcome reached statistical significance, and both prediction intervals included the null, indicating that the magnitude and direction of these associations may vary across clinical settings. Importantly, neither was associated with increased graft loss. Both infection and acute rejection have, however, been independently associated with prolonged hospitalization after LT, while higher MELD scores at transplantation have also been linked to increased LOS [55, 56]. The longer hospitalization observed in our study is therefore likely multifactorial, and preventing complications such as infection and graft rejection may help reduce LOS after eLT.

This increased resource utilization carries substantial financial implications, with eLT costing approximately $300,000 per patient, driven largely by hospitalization and further increased by postoperative complications [57]. These costs must be weighed against the clinical and economic consequences of withholding transplantation. Many patients with corticosteroid-refractory severe AH die without LT, while survivors may progress to chronic liver disease and remain at risk of recurrent episodes, further hospitalizations, and complications [58]. In this context, the cost of eLT may represent less an additional expenditure than a reallocation of the substantial resources otherwise required to manage advanced ALD [59].

### Limitations

Several limitations should be considered when interpreting these findings. Ten of the twelve included studies were retrospective cohorts, with the remaining two comprising a non-randomized trial and a prospective cohort with historical controls [14, 25]. None was judged low overall RoB, with confounding representing the principal concern. Early LT recipients were also consistently more acutely ill at baseline, suggesting that any residual confounding would likely bias results against eLT. Follow-up duration varied considerably across studies, which is particularly relevant for time-dependent outcomes such as alcohol relapse and mortality, although for the latter this was partly mitigated by the analysis of patient survival [18].

Heterogeneity was observed across several outcomes. In the LOO analysis, the main outlier for infection was Legg et al., in which eLT recipients had longer pre-transplant intensive care stay than those in any of the other cohorts, whereas most sLT recipients remained at home before transplantation [29]. For alcohol relapse, heterogeneity likely reflects differences in follow-up duration and addiction management across studies. Recidivism was variously assessed through interviews, biomarkers, or both, with some studies requiring or providing addiction treatment before LT [14, 24, 25].

For LOS, the main outlier was Fu et al., the only cohort in which the two groups were comparable across most baseline characteristics [26]. In three studies, the mean LOS had to be estimated from medians and interquartile ranges, with the underlying data skewed in one or both groups [23, 26, 29]. Together with differences in clinical presentation and institutional management, these methodological variations may partly explain the heterogeneity observed for this outcome.

Finally, funnel plot asymmetry should not be interpreted as definitive evidence of publication bias. Selective outcome reporting remains possible, as any alcohol relapse, acute rejection, and infection were each reported by only five or six cohorts. The most asymmetric plots, for relapse and infection, were also those with the highest I² values, suggesting that true between-study heterogeneity may contribute substantially. Small-study effects appear less likely, as in both plots the asymmetry was driven from Legg et al., the second-largest cohort contributing to these outcomes, whose position on the funnel plot reflects a low number of events rather than a small sample size.

## Conclusions

Early LT was associated with survival outcomes similar to those of standard LT for ALD, challenging the survival-based rationale for a fixed 6-month abstinence period. Evidence regarding alcohol relapse is less conclusive, as statistical significance emerged only in sensitivity and subgroup analyses, while no corresponding increase in graft loss was observed. Given the longer hospitalization and associated resource utilization seen with eLT, our findings support rigorous multidisciplinary candidate selection incorporating both clinical and psychosocial criteria, together with close post-transplant surveillance, particularly among recipients with AH. Instruments such as SIPAT and the SALT score may provide a structured framework for psychosocial assessment, while standardized monitoring incorporating objective biomarkers such as PEth may improve post-transplant relapse detection and enable prompt intervention.

## Supplementary Information

Below is the link to the electronic supplementary material.

Supplementary Material 1 (DOCX 270 KB)

Supplementary Material 2 (DOCX 2.49 MB)

## Data Availability

Raw data and the analytic code in Rstudio can be provided upon request to the corresponding author.
