# Supplementary material for: Clinical outcomes of early versus standard liver transplantation in patients with alcohol-related liver disease: a meta-analysis
Source: Langenbecks Arch Surg. 2026 Oct 2;411(1):266. doi: 10.1007/s00423-026-04310-0 (PMC13645987; doi:10.1007/s00423-026-04310-0)
Supplement: Supplementary file 2 [file 423_2026_4310_MOESM2_ESM.docx]

Supplementary material

("Alcohol Liver Disease" OR "Alcoholic Hepatitis" OR "Alcoholic Liver" OR AALD OR "Severe Alcoholic Hepatitis" OR "Severe Alcohol Hepatitis" OR "Alcohol Liver" OR "Alcohol Associated Liver Disease" OR SAH OR AC OR AH) AND ("Liver Transplantation" OR Transplantation OR LT OR "Liver Transplant") AND (Early OR Standard OR "Early Transplantation" OR ELT OR "Early Liver Transplantation" OR Premature OR Preemptive) AND (Late OR "Late Transplantation" OR Standard OR Delayed OR Abstinence OR "6-months" OR Sobriety OR Sober)

Supplementary table 1. Full search algorithm of the study.

Supplementary figure 1. Forest plots from the leave-one-out (LOO) sensitivity analysis; RR= Risk ratio; MD= Mean difference; CI= Confidence interval; eLT= Early liver transplantation; sLT= Standard liver transplantation.

Supplementary figure 2. Forest plots of the pooled outcomes restricted to patients with alcoholic hepatitis in the intervention group; RR= Risk ratio; HR= Hazard ratio; CI= Confidence interval; eLT= Early liver transplantation; sLT= Standard liver transplantation; IV= Inverse of variance; MH= Mentel-Haenzel;

Supplementary figure 3. Contour enhanced funnel plots for publication bias assessment.
